# Navigating silence: cultural, familial, and immigration influences on the sexual violence experiences of Asian female college students in the university of California system

**DOI:** 10.1186/s12889-025-24487-1

**Published:** 2025-10-01

**Authors:** Jianchao Lai, Eunhee Park, Jenny Lee, Rhea Gandhi, Jennifer Wagman

**Affiliations:** 1 Department of Social Welfare, Luskin School of Public Affairs, University of California Los Angeles, 337 Charles E Young Drive East, Los Angeles, CA 90095 USA; 2 Department of Community Health Sciences, Fielding School of Public Health, University of California Los Angeles, Los Angeles, USA; 3 School of Medicine, University of California Irvine, Irvine, USA

**Keywords:** Sexual violence and harassment, Asian female college students, Cultural stigma and family dynamics, Help-seeking behaviors, Immigration and racial discrimination

## Abstract

**Background:**

Sexual Violence and Sexual Harassment (SVSH) on college campuses disproportionately affect racially minoritized groups, including Asian female students. These individuals face unique cultural and familial challenges impacting their experiences and help-seeking behaviors. Existing literature highlights barriers such as cultural stigma and the model minority myth, but research specific to this population is limited.

**Objectives:**

This study explores the SVSH experiences of Asian female college students and examines how cultural norms, family dynamics, and immigration background influence their responses and access to support services.

**Methods:**

Data were collected from all 10 University of California campuses as part of the Double Jeopardy (DJ) project. Fifty-one semi-structured interviews were conducted. A grounded-theory-guided thematic analysis identified key themes, with reflective memos used to capture insights and minimize bias.

**Results:**

Findings reveal that cultural norms and family expectations shape Asian female students’ responses to SVSH, often reinforcing self-blame and silence of the incidents. Immigration background added complexities, with fears related to legal status and unfamiliarity with SVSH frameworks and existing support services in the United States (U.S.). Mental health stigma and language barriers further contributed to isolation and reluctance to seek help. Despite challenges, participants expressed the need for culturally competent, linguistically appropriate resources.

**Conclusions:**

Universities must adopt culturally competent policies, expand multilingual counseling services, and collaborate with community-based organizations to support Asian female survivors. Addressing systemic barriers can foster an inclusive environment that empowers survivors and enhances access to necessary resources.

**Supplementary information:**

The online version contains supplementary material available at 10.1186/s12889-025-24487-1.

## Introduction

Sexual violence is a highly pervasive public health issue, particularly on college and university campuses, impacting nearly 1 in every 5 female-identifying students [1]. Little is known about culturally appropriate and accessible services for racial minority student-survivors of sexual violence and sexual harassment (SVSH), who have been found to be disproportionally impacted [2–4]. Since the start of the COVID-19 pandemic and stay-at-home orders, the risk for SVSH was increased due to isolation, particularly intimate partner violence (IPV) [5]. 

Intersecting factors make sexual assault increasingly prevalent on college campuses [6]. Survivor-focused research has identified effective training to be lacking in higher education, leading to poor understanding of what qualifies as SVSH and under-utilization of resources [7]. This is compounded by the lack of physical and mental healthcare resources, preventative campus-wide initiatives, and a large-scale fear of reporting to the institution [8–11]. Help-seeking barriers are often magnified for Asian survivors, who navigate a unique sociocultural landscape that impacts their help-seeking behaviors and hinders their experiences accessing support systems. The “model minority” myth fosters a damaging narrative that downplays their need for assistance while intensifying societal and familial pressure to prioritize external expectations over personal well-being [12–14]. Coping mechanisms shaped by cultural norms and intergenerational dynamics can further complicate healing journeys, leaving many survivors without adequate resources [15]. 

To address these gaps, it is critical to examine the social, historical, and cultural factors that influence the lived experiences of Asian survivors [16, 17]. This study situates the experiences of Asian female college student victims/survivors within a broader context, exploring how factors such as anti-Asian discrimination, gendered microaggressions, and xenophobia intersect with histories of immigration and cultural identity to impact individuals’ journeys to SVSH disclosure and recovery. These complex influences underscore the importance of understanding the unique challenges facing this population, as informed by the historical and cultural narratives that shape their social realities.

## Literature review

### Asian college students and SVSH

More than half of women have experienced SVSH in their lifetime, in particular women of racial and ethnic minority backgrounds carry the highest burden [18]. 39% of non-Hispanic American Indian women, 30% of non-Hispanic Black women, and 15% of Latinx women report having experienced SVSH in their lifetime [19]. Despite the known statistics, there remains little research for Asian women in relation to SVSH. In the few studies that captured this information, reporting among Asian American women was one of the lowest. This largely stems from limited understanding of SVSH, high acceptance of rape myths—false beliefs about sexual assault—and negative attitudes toward sexual violence survivors^1^.(19,20) One specific factor which often is described as the “othering-based stress” (also known as minority stress) is found to be positively associated with SVSH among minorities as it impacts coping mechanisms and help-seeking behaviors. This type of stress, exhibited by individuals who are seen as “different” through stigma and distancing, requires an individual who is “othered” to navigate situations with additional effort [20]. Among women of color, experiences of race-based discrimination is intrinsically tied to sex-based discrimination. Intersectionality, a framework coined by Kimberle Crenshaw, explains the compounding effects of racism and sexism when they interact, reinforcing one another [21]. The impacts of intersectional discrimination on Asian women, while underexplored, is best explained under the lens of U.S. imperialism. Historically, Asian women have been shaped by the intersection of Orientalism and sexism due to their role as sex workers. They are typically described as a “dragon lady” or “China doll”, illustrating their sexual tropes as either hypersexual and deviant or desperate and subservient, respectively. These images made way for sexual objectification and furthermore, racialized and gendered violence against Asian women [22]. 

Students on college campuses are highly at risk for sexual violence. This risk is exacerbated through decreased parental presence, increased alcohol use, increased sexual activity, and exposure to peer norms [23]. Females 18 to 24 year old have the highest rate of sexual violence victimization compared to females of other ages, with 81% of victims experience rape before age 25 [23]. Although Asian and Pacific Islander students appear to be at the lowest risk when viewing data by race/ethnicity [23], reporting relevant statistics for this demographic be potentially biased due to certain unique challenges raised by their culture. This difficulty stems from influence of family culture, shame and stigma from peers, and the role of media and educational opportunities [24]. Family often reinforced their beliefs in SVSH victims having to bear the responsibility of their experiences, coupled with limited communication between their parents/guardians [24]. To fill these gaps, students often turn to the internet or social media to understand SVSH, identify signs of abuse, and seek guidance on self-care strategies [25].While digital resources can be empowering, they also carry the risk of misinformation, particularly in the absence of culturally and linguistically appropriate education [25]. 

Furthermore, the different types of harassment and violence also had varying degrees of internalization among this demographic. It is easier for Asian students to recognize overt and obvious types of harassment, such as physical, rather than verbal and visual sexual harassment (e.g., being shown a pornographic image without consent, obscene jokes). Particularly, Asian international students often felt confused on terminology and the concepts of Title IX. Training and workshops on SVSH often overlook these potential challenges among non-English speakers, instead of turning them into opportunities (e.g., workshops in cultural groups, conducting the workshop in their native language) [24]. The culture of silence, limited SVSH education, and the stigma around disclosure contribute to persistent underreporting among API college students and reflect a broader gap in the literature [26]. Grounded in intersectionality theory, these unique social factors highlight the need to examine Asian students’ SVSH experiences through the lens of overlapping identities—including race, gender, and immigration status—that shape both their risk of victimization and their access to support.

### Asian cultural values

Eastern and Western culture both play a large role in understanding an individual’s role and expectation in relation to their family, which impacts their ability to identify, report, and cope with SVSH [27]. Asian culture values harmony with the external environment, meaning an individual must prioritize conforming and the greater collective – as opposed to an individualistic and tunnel-visioned mindset [27]. Confucianism explains many of these ideologies as it informs many Asian principals of human relations, social structures, and governance [28]. To preserve a harmonious family, shame, self-restraint, and suffering are all expected of Asian family members as the needs of the group precede those of an individual [28]. For example, Asian families may be more reluctant to disclose a SVSH incident as it may be shameful and does not benefit the collective [27]. As such, the individual is expected to “suffer in silence” to preserve the family image. The family structure is shaped by gender and age hierarchies in which elders and males hold a higher status in the family as opposed to younger individuals and females [27]. In line with patriarchy, Asian families expect girls are raised to be subordinates, while boys are educated to exert power [28, 29]. A woman’s husband, father, and son are all central to her identity [29]. This is described as filial piety, and in some research seen as a protective factor as theoretically people practicing should avoid familial conflict at all costs to preserve unity [29]. Whereas Western culture may revere an individual’s initiative and assertiveness, Asian culture expects an individual to stay away from standing out and instead follow the middle-position virtue – the ability to blend in with others and become inconspicuous [29]. This virtue, along with the hierarchies experienced by Asian family members, makes it difficult for women to report SVSH and place Asian women in particular, at risk [27]. Members of Asian households have become skilled at avoiding dissonance within the family using “loss of face”, a social concept of shame, as a method of enforcement [27]. 

### Effects of Asian immigration history and the model minority myth

Immigration from Asia to the United States (US) was highly conditional and served as the basis for what we now describe as the Model Minority Myth (MMM). The first individuals to emigrate to America were from China. While the first recorded Chinese individuals came as early as the late 1700 s for trade and educational missions, gold in California attracted large numbers of Chinese immigrants in the mid to late 1800 s [30]. Immigrants from Japan had also moved to Hawaii for opportunities to work in the sugar plantations [30]. As a result of the overwhelming influx of Asian immigrants, the US government began to pass laws to restrict their freedoms and dissuade immigration [31]. The Alien Land Law of 1913 prohibited immigrants from owning and leasing land in California, while the Immigration Act of 1917 barred Asian Indian immigrants specifically from entering the United States [31]. The Chinese Exclusion Act of 1882 limited admission of unskilled workers, with the Gentleman’s Agreement extending this provision to Korean and Japanese immigrants [30]. One way to circumvent these restrictions was through the Immigration and Nationality Act of 1965, which allowed individuals with professional backgrounds or specialized skills to immigrate to the US [31]. This sparks the beginning of the MMM, in which Asians living in the US are viewed as educated professionals and “model” citizens.

On the exterior, many Asian immigrants saw the concept of “model citizen” as a source of their pride – an indicator of assimilation and no longer feeling “othered” [31]. Under the surface, it also encouraged silencing of problems and conflict with the dominant culture as a means to meet American expectations [31]. Acculturation is a large factor of MMM and how Asian Americans present themselves in order to assimilate and achieve upward mobility. Both highly influence Asian help-seeking behaviors and reporting rates of sexual violence. Although few have explored this specifically in the context of Asian female college students, studies have shown that Asian immigrant women are less likely to report abuse or receive preventive care and treatment services due to numerous barriers (e.g., culture, language, institutional) [28]. Kim and Lee conducted a quantitative study and explored the relationship between help-seeking attitudes, internalized MMM, and Asian values among Asian American undergraduate students. The results showed a statistically significant correlation between internalized MMM and health seeking attitudes among students, and the MMM stereotype served as a barrier to help-seeking attitudes [25]. This demographic’s lack of utilization of formal services (e.g., women’s shelters, police, hospitals) are also impacted by their internalization of patriarchal norms and their desire to avoid conflict [28]. Immigration and the internalization of MMM plays a large role in shaping one’s response to sexual violence, particularly for Asian women.

### The impact of historical and intergenerational trauma in Asian families

Historical trauma and intergenerational trauma, although used interchangeably by many, have very different meanings. Historical trauma refers to the political structures that oppress indigenous, immigrant, and other marginalized groups, effectively perpetuating health disparities [32]. 

War, political turbulence, and scarcity of resources were root causes for the wave of migration from Asia to the United States [32]. These events left lasting wounds on individuals both mentally and physically, but also between Asian American families across generations. Instances in which families communicated about these traumatic experiences were few and far between [32]. Silence to avoid discussion negatively impacts the Asian parent-child relationship, especially because trauma is typically used during conflicts or arguments to shame and discipline children [32]. For example, parents who allude to their trauma in reference to the privilege and ease their children have in the United States [32]. One study observed Japanese Americans parents and their conversation with their parents surrounding internment camps. Withholding sharing as an attempt to protect their children, often resulted in loss of identity, family history, and self-consciousness of a child’s ethnicity [33]. This use of historical trauma manifests into intergenerational trauma, when it is passed onto the next generation in a way that does not promote positive outcomes [32]. On the other hand, communication through conversations, storytelling and shared narratives can allow space for collective resilience [32]. These types of communications typically affect connectedness between parents and children, as well as children’s emotional well-being [33]. There has been little research done on how these types of trauma relate to SVSH outcomes among Asians. However, silence as a practice of avoidance and a result of shame, discourages Asian individuals from speaking out.

### Research gap and aims

While extensive research exists on sexual violence against college students, there remains a significant gap in understanding the specific experiences of students from racially minoritized groups, particularly Asian female student-survivors^2^. Research exploring the unique cultural, familial, and immigration-related challenges faced by this population remains limited.

This study seeks to address these gaps by exploring the SVSH experiences and help-seeking behaviors of Asian female college students, with a particular focus on the intersection of Asian culture, Asian family values, racial discrimination, and immigration background. By examining how these factors influence both the survivors’ experiences of SVSH and their engagement with campus and external support systems, this research aims to provide insights that can inform more culturally responsive policies and services, ultimately improving support for Asian female survivors within university settings.

## Methodology

### Data collection

The current study, as a part of the Double Jeopardy mixed-method project, recruited participants from all 10 University of California campuses, including the solely graduate program at UC San Francisco. The Double Jeopardy Study aimed to investigate the intersection of anti-Asian racism and sexual violence/sexual harassment (SVSH) among ANHPI female college students. Through mixed methods—including survey, qualitative interviews, and transmedia-photovoice—the original study examined mental and physical health outcomes, help-seeking behaviors, institutional responses, and culturally relevant service needs of this population in the context of COVID-19. The subset of interview participants included in this study self-identified as Asian women who were current or recent (within the past year) undergraduate or graduate students at one of the ten University of California (UC) campuses. To be eligible, participants must have reported experiencing sexual violence and sexual harassment (SVSH) while enrolled at a UC campus.

The design and data collection methods were described in detail previously [34]. Participants who completed the survey and indicated a willingness to engage further were contacted via email to schedule an interview. The demographics of the interview sample are presented in Table 1. At the beginning of these interviews, the researchers revisited the consent forms with each participant to address any questions and ensure understanding of the study’s scope and their rights. This process was designed to ensure a thorough understanding and comfort for the participants, reflecting our commitment to ethical research practices. The research protocol received full approval from UCLA’s Institutional Review Board (IRB#21–000149).

**Table 1:** Demographics of interview participants (*N* = 51)

	*N*	%	Range
Race			
* Asian-alone*	46	90.20	
* Mixed-Asian*	5	9.80	
Asian ethnicities			
* Chinese*	26	50.98	
* Japanese*	3	5.88	
* Indian*	7	13.73	
* Korean*	4	7.84	
* Indonesian*	2	3.92	
* Vietnamese*	6	11.76	
* Laotian*	1	1.96	
* Filipino*	9	17.65	
* Mongolian*	2	3.92	
UC Campus			
* UC Berkeley*	3	5.88	
* UC Davis*	3	5.88	
* UC Irvine*	2	3.92	
* UC Los Angeles*	31	60.78	
* UC San Diego*	3	5.88	
* UC Santa Barbara*	6	11.76	
* UC Santa Cruz*	2	3.92	
International Student			
* Yes*	14	27.45	
* No*	37	72.55	
Transfer Student			
* Yes*	5	9.80	
* No*	46	90.20	
Types of SVSH Experienced			
* Sexual harassment*	38	74.51	
* Intimate partner violence/Dating violence*	17	33.33	
* Stalking*	10	19.61	
* Sexual assault*	27	52.94	
* Other*	2	3.92	
Sexual Orientation			
* Bisexual*	16	31.37	
* Homosexual*	3	5.88	
* Not sure/refused to answer*	6	11.76	
* Queer*	1	1.96	
* Pansexual*	2	3.92	
* Heterosexual (Straight)*	23	45.10	
Age			(19, 32)

The interviews, conducted in English, Chinese, or Korean—reflecting the high number of students from China and South Korea in 10 UC campuses—were designed to delve into barriers to seeking SVSH- and health-related services, factors influencing help-seeking behaviors, the impact of COVID-19 on these processes, and culturally appropriate strategies to facilitate access to necessary services. The interview guide allowed for probing and expansion of topics as needed, and interviewers, trained in trauma-responsive approaches, prioritized participant well-being throughout the process. Participants were reminded of their rights at the beginning of the interview, offered the use of a pseudonym, and could request breaks or skip questions. Each interview segment started with an overview of the upcoming topics, including content warnings when appropriate. The interview data was collected over Zoom between October 2021 and November 2022 by a team of five UC student researchers who were trained in trauma-responsive approaches. Reflective practices such as pre/post-interview memos facilitated ongoing discussion within the research team, ensuring a sensitive and thorough exploration of the difficult topics at hand. Participants were compensated with a $50 e-gift card and provided with a list of SVSH advocacy services, underscoring our commitment to their welfare beyond the study’s scope. All interviews were audio-recorded with consent, and any identifiable information was anonymized or removed during transcription to protect against disclosing any identifiable information.

### Data analysis

Our analytic approach was grounded in Braun and Clarke’s theoretically flexible framework for thematic analysis, while drawing from grounded theory strategies to strengthen theoretical sensitivity [35, 36]. Specifically, we applied grounded theory principles such as constant comparison—revisiting earlier transcripts as new codes emerged to ensure analytic consistency—and maintained an emergent design by iteratively refining our codebook throughout the process. Guided by this approach, we adopted an inductive, bottom-up orientation to theme development, allowing categories to be built from participants’ lived narratives rather than a priori assumptions, while remaining mindful of our research aims [36]. Each interview transcript was generated using the Trint transcription service, which operates on Amazon AWS servers; the data were securely stored in encrypted AWS S3 buckets. After completing the transcription process, we conducted preliminary coding to discern central themes and patterns within the data by randomly selecting 25% of the transcripts. This phase involved meticulous line-by-line open coding to pinpoint and label emerging concepts. Memo-ing was utilized consistently during each phase of the analysis to capture the researchers’ reflections, biases, and insights, enriching our understanding of the data. These memos played a crucial role in shaping a conceptual framework during discussions with our 8-member research team.

Following the initial coding phase, we arranged the preliminary codes into key themes and then applied the semi-finalized coding tree to the remaining transcripts, progressively refining our coding structure as analysis advanced. Our objective was to reach saturation, a point at which no new themes emerged from further coding and analysis. Ultimately, this rigorous analytical process culminated in the formulation of a theoretical model that encapsulates a range of SVSH experiences within the Asian student community, highlighting their help-seeking behaviors and the obstacles they encounter in accessing support services.

### Positionality statement

The authors recognize that their social identities, backgrounds, and lived experiences informed their engagement with this research throughout. As members of the Double Jeopardy Project research team, which includes undergraduate and graduate students from a variety of disciplines, the team brings shared experiences and identities that closely align with those of many study participants. These include, but are not limited to, identities as survivors of sexual violence, international students, and students of color. These connections informed the study’s design, the development of culturally relevant research tools, and the interpretation of findings, grounding the research in both academic rigor and personal insight.

## Results

**Fig. 1:**
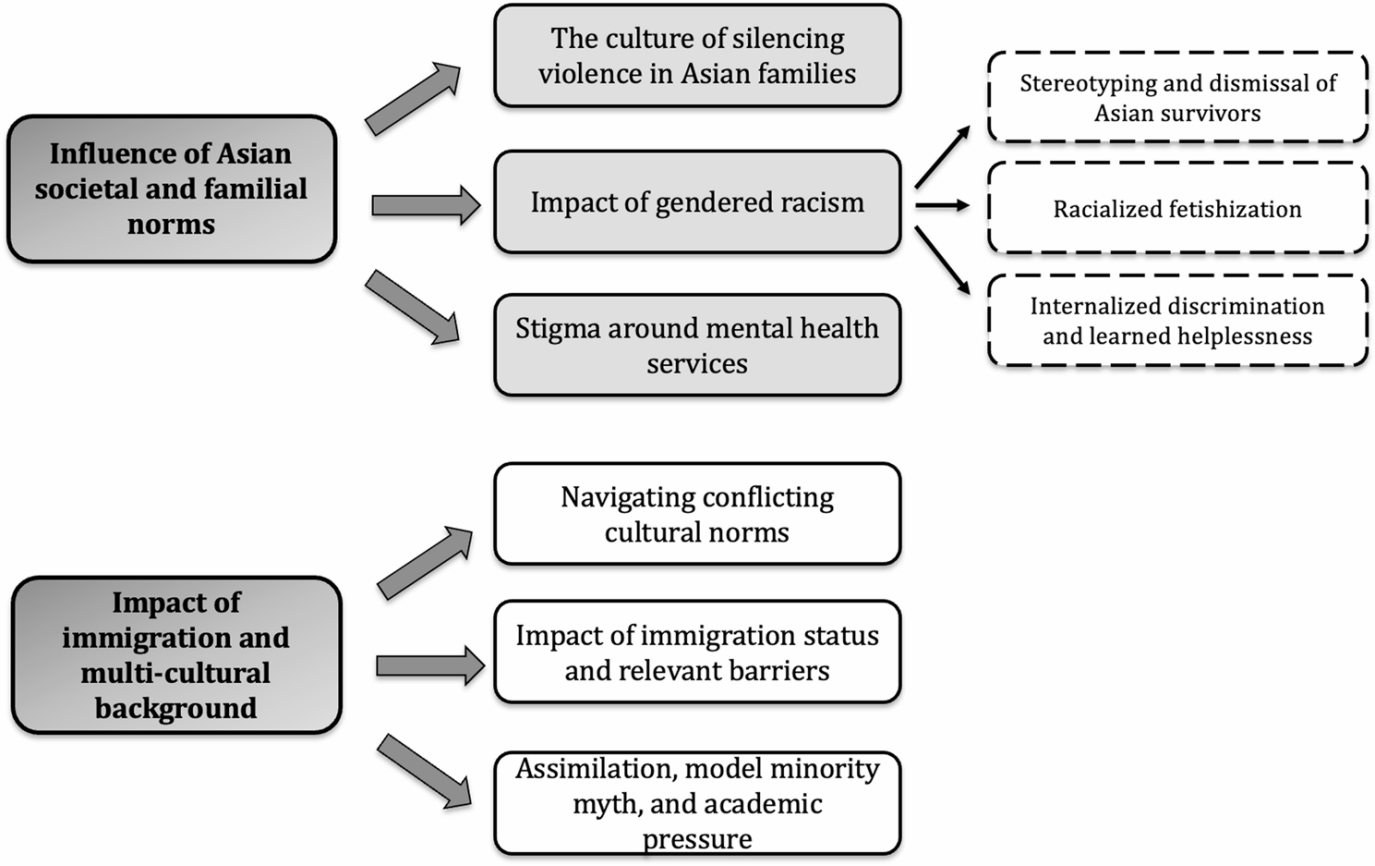
Qualitative themes and subthemes on Asian students’ experiences with SVSH

The cultural context and family immigration history play significant roles in shaping the experiences and perceptions of sexual violence, sexual harassment, and sexual health (SVSH) among Asian female students. The result explores the impact of cultural norms, family immigration history, social expectations, and a variety of unique challenges faced by these students, particularly within the perceived Asian cultural framework. A visual overview of the emergent themes and their structure is presented in Fig 1.

### Influence of Asian societal and familial norms

#### The culture of silencing violence in Asian families

Asian family culture played a crucial role in perpetuating cultural stigma and shame surrounding SVSH. This theme captured how cultural norms and familial expectations around shame, obedience, and honor contribute to the silencing of SVSH experiences. These deeply rooted values often discourage disclosure, foster internalized blame, and create substantial barriers to seeking support. One participant shared, “*We were taught to put the blame on ourselves or to reflect on our mistakes before we put that responsibility toward the other person*,* even though that person obviously assaulted us or disrespected us.”* This reflected a common pattern identified from the interviews where survivors were socialized to suppress and normalize harm.

Participants often stated that their families, especially female relatives, minimized the significance of Intimate Partner Violence (IPV) and other types of SVSH, discouraging them from seeking help. One participant recounted, “*When I told my aunt about the abuse*,* she said it was just normal relationship problems and that I should be patient.”* This minimization can perpetuate a cycle of abuse, as survivors were led to believe that their suffering is insignificant or a “*normal part of life*”. The interviews also highlighted the significant influence of female family members in perpetuating cultural stigma and shame surrounding sexual violence. One participant mentioned, “*My mother always told me to keep quiet about such things. She said it would bring shame to our family.”* Another shared, “*In my family*,* we don’t talk about these issues because it’s considered shameful. My grandmother would always remind us to keep such matters private to maintain our dignity.”* These statements illustrated how deeply ingrained cultural norms and expectations from female family members contributed to the silence and stigma surrounding SVSH incidents.

Participants also described how cultural values emphasizing family harmony and avoidance of conflict influenced how SVSH experiences were handled within the home. In situations involving intrafamilial perpetrators, survivors and their family members often chose silence over confrontation, especially when the perpetrator held a close relational tie. One participant reflected on her experience of being sexually assaulted by her uncle:

*“…for [the SVSH incident] with my uncle*,* because it’s like her [participant’s mother] brother*,* so she [my mom] basically like… we just have reached an equilibrium where we don’t talk about it*,* and I have learned to do all the things that I need to deal with.”* – Undergraduate, Filipino, Asian American student.

This narrative revealed a form of emotional compartmentalization—a coping strategy where the survivor adapts to silence as a means of preserving the broader family structure, even at personal cost. Rather than address the abuse directly, the family appeared to adopt a stance of passive endurance, reinforcing silence as a form of survival and loyalty.

This tendency to minimize or rationalize harm was not limited to SVSH but extended to other forms of interpersonal violence within the family. Participants described how authoritarian family dynamics and expectations of obedience often silenced dissent or critique, even when a parent was abusive. One participant shared:

*“I don’t like it when dad is like that [domestic violence behaviors]and she [the mother] just kind of like justifies it—’just have a listen to what he says and then you just obey him and then things will be like fine[…] or like he doesn’t really mean the things like he did and like*,* I don’t really—she*,* she won’t like really say that the things that he did is like wrong but she also like*,* I don’t know*,* I think she kind of understands that she is like also like in denial about it and she thinks it’s easier to stay and to work out a different solution.”* - Undergraduate, Chinese, International student.

These experiences illustrated how intersecting dynamics of gendered obedience, denial, and cultural scripts about preserving family unity can suppress disclosure and impede emotional validation for survivors. Silence, in this context, becomes both a learned behavior and a protective mechanism in families navigating cultural expectations and unresolved harm. The interview data also reflected a common coping mechanism within the family, where problematic behaviors were rationalized or dismissed to maintain harmony, often at the expense of confronting the underlying issues. In other situations, it could also be the survivors who were trying to protect their family by not disclosing their SVSH experience, as one described their experience: “*because one of my grandmother also got diagnosed with cancer. So they were also dealing with their own issues…so I didn’t want to burden them with more things.”*

Cultural norms heavily influenced perceptions of relationships and personal boundaries. Growing up in a “*conservative”* Asian household often meant that topics related to sex and relationships were taboo. Participants reflected that there was a lack of understanding and communication about healthy relationships and boundaries. Participants expressed that the heavy stigma surrounding sexuality led to significant mental punishment and social pressure, making them feel isolated and out of place within their home. While not always referencing specific SVSH experiences, these accounts contextualized the cultural silencing that shaped how Asian youth were taught to view sexuality and autonomy. One participant stated:

*“Um*,* and it came it came to me from a very early age*,* almost in my teenage years*,* where I was told or I was punished by my mom if I wear if I wore a certain kind of clothes*,* um*,* stand or sit too close to men*,* or even discuss questions about my body parts*,* which I genuinely did not know and wanted to have that kind of knowledge… but the stigma of sexuality is so heavy that the mental punishment or social pressure really push me to a corner that I feel that I might be one of the few that do not belong to the culture.”* – Undergraduate, Vietnamese, Asian American student.

#### Impact of gendered racism

##### Stereotyping and dismissal of asian survivors

Exposure to gendered racial discrimination over time played a significant role in shaping study participants’ SVSH disclosure and recovery processes. Participants described recurring experiences with racialized stereotypes that positioned Asian women as passive, compliant, or unlikely to resist mistreatment. These was a common stereotype experienced by study participants that people perceived Asian women do not stand their ground or speak up against injustices. Study participants shared that this internalized racial discrimination led to dismissive attitudes toward their own experiences of racism and SVSH. One participant reflected on this perception, recalling remarks like, “*You guys don’t even experience racism. You are paid just as much as white people. You shouldn’t be complaining.*” Such comments reflected a broader societal tendency to dismiss Asian experiences with racism, particularly when those experiences didn’t fit dominant narratives of marginalization. While sometimes not directly referencing SVSH, this erasure of racial discrimination shaped an environment in which survivors felt their broader struggles with dismissal and invalidation, including those related to SVSH vulnerability and help-seeking, were minimized or not taken seriously. The downplay of racism against Asians contributed to the participants’ expectation that their own SVSH-related struggles were not acknowledged, minimizing the real challenges they faced.

##### Racialized fetishization

Participants’ observation of “*Asian fever*” pointed directly to another problematic aspect of racism against Asian women: fetishization. This term described the exoticization and objectification of individuals based on their racial or ethnic characteristics. Participants described scenarios where one was attracted to another solely because one’s racial features, reducing the person’s identities to an overly simplified stereotype. The experiences of Asian victims/survivors of SVSH revealed the layers of objectification and fetishization that stem from racial profiling. One participant shared:

*“I also realize a lot of things*,* such as they have what they call ‘Asian fever.’ Some of them explicitly admitted that they love being with Asian women because I always look like a teenager*,* even though I am in my early thirties.”* - Undergraduate, Vietnamese, International student.

*“Woman who has like an East Asian identity in society*,* I’ve experienced plenty of like*,* men who fetishize Asian women*,* and I’ve experienced a lot of that”* - Undergraduate, Mixed-ethnicity (Chinese, Indian), Asian American student.

##### Internalized discrimination and learned helplessness

Across the interviews, internalized discrimination and learned helplessness emerged as powerful and pervasive themes. Participants often expressed a deep-seated belief that they were undeserving of support due to societal perceptions. For instance, one Vietnamese student shared, “*I’ve always felt like I don’t deserve help because of how society views us Asians*.” Similarly, another undergraduate participant, identified as mixed-ethnicity (Chinese, Japanese) Asian student, articulated the profound impact of this internalization: “*I’ve internalized so much negativity about being Asian that I often feel helpless and don’t believe things will change.”* This pervasive sense of helplessness was a recurring barrier, actively preventing many victims and survivors from seeking the support they needed.

#### Stigma around mental health and mental health services

Participants discussed the cultural stigma against seeking mental health and relevant services, particularly within East Asian communities. Participants explained how East Asian culture is perceived to be *“not expressive”* and tend to be *“very subtle.”* They highlighted how crying and open emotional expression were seen as unacceptable, leading individuals to internalize their feelings. This cultural norm affected their experiences with therapy, as they struggled to express emotions openly even when feeling upset or depressed. One participant shared their experience of trying to find a therapist to cope with her intimate partner violence incident:

*“I’ve had times in therapy with white therapists… where I haven’t like*,* I’ve like been really upset and I’m like*,* Oh*,* and like really feeling this right now. But I’m not to the point of crying and just letting all my emotions out in front of someone. And that’s just not how I grew up with my parents.”* - Undergraduate, Mixed-ethnicity (Indian and Chinese), Asian American student.

This cultural stigma and fear of judgment contributed to participants delaying seeking professional help. One participant explained their perception of different help-seeking resources:

*“I had support through my friends…I didn’t really like ever talk about it with my family because*,* being Asian*,* it’s so weird to bring that up. I started to go to therapy for it at [university campus]*,* but previously I didn’t go to therapy for it because this is kind of weird to talk about in general*,* like even to bring up to my family*,* let alone like a therapist. I wish I’d gone [to therapy] earlier.”* – Undergraduate, Mixed-race (White/Asian), Asian American student.

This issue was further reinforced by the family attitudes, where many of these students grew up with parents who did not acknowledge or believe in mental health issues, creating an environment where discussing feelings was not perceived as “*safe*”. Student survivors mentioned how there was “*no vocabulary”* for mental health in these families meant that complex emotions and mental health struggles were often dismissed as either *“crazy*” or nonexistent by their families. This lack of understanding of mental health often was attributed to the Asian cultural norms. One participant noted, *“In our culture*,* we don’t talk about these things [mental health issues] openly. It’s considered taboo.”* The lack of open dialogue about these issues often left survivors to navigate their trauma alone, without the necessary familial support that could aid their healing process. Some participants described how Asian culture often reflected a general distrust of mental health and relevant services, which frequently resulted in family members withholding support for mental health concerns:

I am super open about like different things and I don’t mind talking about them, but I’m not generally open about mental health or sex. My mom found out, I was back in 11th grade, that I was depressed…I took a depression test online and I checked all the boxes. I was depressed, and I was also practicing self-harm. My mom found out, but she didn’t know what to do about it… And we brought it up in a dinner conversation once and we never talked about it ever again. I feel like mental health issues in my family kind of just slide by like, just get through it, but you get through it like, it’s like not discussed….

*– Undergraduate, chinese, Asian American student*

“Because as I grew up, I was always sort of doing things alone because, you know, like immigrant parents, they didn’t really understand everything that, I think, they don’t understand, like mental health issues or things like that. They don’t really believe in them.”

*— Undergraduate, indian, Asian American student*

“Participants also highlighted the broader cultural emphasis on self-reliance, with one stating, *“Asian culture is kind of just to like*,* deal with things yourself*,* like not be openly expressive about a lot of things.”* This reinforced the idea that emotional self-management was a valued trait, further discouraging open discussions about consent and emotional needs.”

### Impact of immigration and multi-cultural background

#### Navigating conflicting cultural norms

Participants described the complexity of navigating different cultures, across ethnic identities, intergenerational, and cross-national cultural differences. While this theme may not appear to directly address SVSH experiences, participants’ experiences navigating multiple cultural norms played a critical role in how they interpreted social interactions, recognized harm, and made decisions about SVSH disclosure. Participants who identified as mixed-race shared their internal conflict about belonging, stating, “*Being mixed*,* I feel like I don’t completely belong there like some people can speak the language fluently and I don’t*,* I can’t really do that*.” They expressed feelings of self-doubt regarding their cultural identity, adding, “*Sometimes I have like a conflict inside of me about who I am as a person and if I’m Filipino enough or if I’m Asian enough*,* because being raised in America*,* it’s kind of like I’ve been whitewashed a little bit.*” This sentiment highlighted the complexity of navigating multiple cultural identities and the challenges of feeling fully accepted in either culture.

Another participant, born and raised in the U.S., described feeling deeply connected to American culture. They shared, “*I was born and raised here so I feel much like American*,* like American culture. I never have been outside the U.S.”* Despite their strong identification with American culture, they also felt a strong sense of pride in their heritage and accommodated to the differences among their family members. A participants further elaborated, “*though I do feel like I have my heritage and I’m very proud of that as well*,* especially*,* being close to my grandparents and having certain traditions and*,* you know*,* hearing different languages at home.*” However, participants often experienced being assumed as foreigners due to their appearance. An undergraduate participant explained, “*it feels still very weird when people assume that … almost as if they see me as a foreigner. I’ve been born and raised here*,* and I speak English. You don’t need to ask me*,* ‘Do you speak English?”* This experience underscored the tension between their strong American identity and the external perceptions that challenged it.

Regarding discrimination, participants experience varied across geo-locations. Larger cities in the states were described as more diverse and inclusive, compared to relatively smaller or more rural areas. One Asian international student remarked on the diversity of their city, sharing, “*a lot of times people don’t know that I’m an international student*,* you know*,* [the city] is so diverse.”* However, some also acknowledged that their unfamiliarity with the culture initially made social interactions challenging. Another participant mentioned, “*I didn’t know how close you can stand after someone*,* like how much distance should be maintained when you’re talking to someone or if you’re standing in line.”* This new cultural environment and norms sometimes prevented them from recognizing potentially inappropriate situations. One of the participants specifically compared party and drug cultures between the US and India, observing, “*Parties are definitely different. I feel in India they’re like a little milder and*,* I guess the drug scene is also like more mild in India.”*

#### Impact of immigration status and relevant barriers

The fear of deportation emerged as a significant barrier to reporting SVSH incidents among Asian female students with an immigration background. This fear often stemmed from concerns about legal vulnerabilities tied to their or their family’s immigration status. One interviewee shared:

“*I was afraid to go to the authorities because I thought they might find out about my family’s immigration status and deport us.”* - Undergraduate, Indian, International student.

This sense of insecurity was echoed by another student:

“*My parents always warned me not to attract any attention to our family because of our visa situation*.” - Undergraduate, Japanese, International student.

For many Asian immigrant students, the fear of jeopardizing their family’s legal standing in the U.S. created a paralyzing tension between seeking justice and self-preservation. As a result, survivors may choose silence over action, believing that reporting could lead to dire consequences beyond their personal trauma. This fear not only discouraged survivors from seeking legal recourse but also limited their access to critical support services, further isolating them in their experiences and exacerbating the emotional and psychological toll of SVSH incidents. One student survivor mentioned how it impacted their help-seeking behaviors:

*“I think I was just real scared about everything*,* like*,* and this I know it sounds very silly right now*,* but a part of me was also like*,* I don’t know*,* I don’t want to get into trouble. I don’t want to get deported*,* or like*,* which is like*,* I know it’s silly now*,* but like I was just very scared when I came in [to report the incident].”* – Undergraduate, Indian, International student.

Another consequence of their immigration status was a limited understanding of racial dynamics, which often made it difficult to recognize experiences of racism. One interviewee remarked, “*I didn’t realize the way people treated me was because of my race until much later.”* Another shared, “*It took me a long time to understand that the microaggressions I faced were actually racial discrimination*.” This delayed recognition can hinder the ability of survivors to understand and articulate their experiences, affecting their help-seeking behavior and their ability to process their trauma. One participant described their hesitancy to label an experience as racism, despite sensing that “*something was wrong*”:

*“I think I was just scared of like naming it*,* like in my head*,* I knew that there was something going on*,* but I was scared of*,* like*,* calling it racism it just felt like a very strong word to use and*,* and I went to therapy about it and like*,* my therapist would make me realize*,* like*,* you know*,* like here*,* like this might be racially motivated like*,* I remember talking to my therapist about this*,* and the first question he asked me was*,* what’s the race of your roommate?”* - Undergraduate, Korean, First-generation Asian American student.

Language barriers were also consistently identified as a major challenge for Asian international students. One participant shared, “*It was hard to find support services that could understand me because I wasn’t fluent in English.”* Another mentioned, “I *felt misunderstood and frustrated because I couldn’t express my feelings properly.”* The lack of language support services could leave survivors feeling isolated and misunderstood, further deterring them from seeking assistance.

#### Assimilation, model minority myth, and academic pressure

For many participants, the pressure to assimilate into U.S. society was not just cultural but deeply personal, manifesting in academic performance, emotional restraint, and silence around trauma. These pressures were magnified by the internalized “*model minority myth*” and high academic expectations placed, particularly those from immigrant families. While these factors may seem distal, participants described how they directly influenced their ability to recognize, disclose, and seek support for SVSH-related experiences. A recurring theme was the emotional burden of academic pressure, which often started early in life and was shaped by parental expectations. As one participant reflected:

*“There was a lot of pressure to perform well academically growing up. Literally my whole life.”* - Undergraduate, Indian, Asian American student.

The student survivors noted the tension between parental values and the realities of college life in the U.S., noting that their families didn’t always understand “*the societal difference between the States and [home country]”*, making their identities harder to reconcile and their pain more difficult to share. The emphasis on external achievement in effort to assimilate into U.S. society often led to learned behaviors of dealing with their own pain in isolation, leaving them unable to disclose their SVSH experiences to even trusted individuals or seek the help they needed. One undergraduate survivor shared:

*“My attitude toward education used to be very extreme*,* almost as the only way of survival. Um*,* and yes*,* of course*,* it works as a huge motivation for me to succeed academically. But the downside of it is I tend to either avoid or put myself out of touch what the reality*,* especially when it comes to interpersonal relationships or most of the time intimate relationship.” -* Undergraduate, Vietnamese, International student.

Participants also shared how stereotypes about being Asian shaped their academic and career trajectories, perpetuating a cycle of pressure to excel. As one participant put it,

*“You have to have straight A’s*,* be a doctor or a lawyer. That mindset was ingrained in me—perform*,* have a high GPA*,* go to a great grad school*,* get a prestigious job.”* - Undergraduate, Indian, Asian American student.

The stress manifested not only through pressure of academic achievements but also through the pressure to appear more “*white.*” Many participants described feeling the need to “*whitewash*” themselves to fit in with their predominantly white peers, which significantly impacted their self-image. Participants who grew up in such communities often constantly compared themselves to white peers. The need to assimilate and gain validation from white peers created a sense of internal conflict and self-loathing, as participants distanced themselves from accepting their identities.

Compounding these struggles was the difficulty in disclosing mental health issues. One participant expressed how they expect oneself to behave when handling trauma:

*“I wanted to be this person who didn’t need anyone’s help*,* who could deal with things on my own.”* - Undergraduate, Mixed-race (Chinese, White), Asian American student.

Even as participants gradually came to recognize that “*there’s nothing wrong with seeking help*,” many still felt it was “*easier*” to keep their struggles to themselves than to explain their SVSH experiences to others. This hesitation in changing help-seeking patterns was reinforced by the cultural and familial dynamics they grew up with, including unresolved personal traumas and dysfunctional family relationships that further complicated their ability to navigate intimate and interpersonal relationships as adults. One participant shared how this mindset impacted her personal life:

*“I was I was almost blinded by the fact that I grew up with a dysfunctional family. So I did not have a good model of how a man should behave and that effects my self-esteem as well. Because my dad keeps neglecting me and I had to constantly try my best by any means to get his approval*,* attention*,* and love…So on the other hand*,* I thought*,* “I have all kind of freedom to date and behave however I want to. But on the other side*,* the fact that the only the endless need for love and the the*,* uh*,* distorted pattern of all concepts about a healthy relationship makes me tend to be in toxic relationship with men who either emotionally manipulated me or even sex solely taken advantage.*

*-* Undergraduate, Vietnamese, International student.

These intersecting factors—including the pressure to meet the expectations of the model minority myth, the emotional strain of assimilation and seeking external validation, and the reluctance to disclose personal struggles—collectively contributed to an isolating and emotionally taxing experience for Asian college students.

## Discussion

The qualitative findings from interviews with Asian female students who were sexual violence survivors illuminated the deeply rooted influence of family culture, immigration background, and racial discrimination on their experiences and help-seeking behaviors. The culture of considering sex as a taboo topic not only prevented survivors from receiving emotional support from their families but also perpetuated ignorance and stigma surrounding SVSH. Participants reported that familial and societal pressures often discouraged them from disclosing their experiences due to concerns about bringing shame upon their families. This finding aligned with Futa et al.’s research, which found that Asian family structures, particularly those influenced by Confucian values, prioritized family honor over individual well-being [23]. Many participants also shared their experience of being directly discouraged by female relatives from reporting, reinforcing cycles of silence and self-blame. Similar to previous studies, this internalized self-blame not only contributed to psychological distress but also acted as a long-term barrier to seeking help or engaging with campus resources [24]. These factors contributed to Asian students’ reluctance to seek support, as many believed they had to manage their challenges alone. This sense of self-reliance was also shaped by their upbringing, during which many felt responsible for helping their first-generation immigrant parents navigate social systems in the U.S. society, which often left them prioritizing their families’ needs over their own well-being. Consequently, they felt isolated in coping with their emotional struggles, particularly when it came to addressing the impact of SVSH victimization.

Immigration status added another layer of vulnerability, particularly for international students, who expressed deep fears of deportation and legal repercussions, which prevented them from accessing formal support systems [37]. This reflected the similar themes noted in Lai et al.’s work, which highlights how international students often had limited awareness of their legal rights under Title IX, compounded by language barriers and a lack of culturally competent services [25]. Many participants felt isolated and unsure of where to seek help, and for some, their immigration status created additional concerns about navigating legal systems. In the absence of clear outreach efforts to proactively inform international students of their rights and protections, survivors were often left to navigate their trauma alone—frequently relying on inaccurate or incomplete information about available resources. Especially in the current political climate, these concerns may be even more pronounced as the shifts in immigration-related policies and public discourse may have heightened the uncertainty and fear among students with immigration backgrounds, potentially exacerbating the help-seeking barriers identified in this study.

Another key barrier to disclosure and support-seeking was the model minority myth, which minimized the struggles of Asian students and falsely assumed they do not experience significant adversity [13–15]. This stereotype obscured the gendered and racialized experiences of Asian female students, particularly in relation to racial fetishization, gendered microaggressions, and heightened academic expectations. In a previous study, Bryant-Davis et al. found that Asian women were among the least likely to report sexual violence, with cultural conditioning and immigration-related fears playing significant roles in their reluctance [37]. These findings further reinforced the need for specialized, culturally informed interventions that acknowledge the unique intersections of race, gender, and immigration status in the experiences of Asian survivors.

To address these barriers, it is imperative for universities to develop comprehensive, culturally competent SVSH policies that are tailored to the needs of Asian students and other marginalized populations. Given that many survivors are unaware of their rights or fear institutional retaliation, universities should implement multilingual outreach programs to ensure that students understand their protections under Title IX. This should include translated materials, culturally specific workshops, and peer-led support programs that make information more accessible and relevant to this population [38]. Additionally, campus counseling services could be expanded to include more bilingual counselors and culturally-trained mental health professionals who can address trauma within the context of Asian familial and cultural dynamics [39, 40]. The stigma surrounding mental health support emerged as a recurring theme in the current study and in prior research, underscoring how mental health concerns are often framed as personal weakness rather than a legitimate need [41]. This finding reinforced the importance of counseling models that are sensitive to the familial and cultural pressures shaping Asian survivors’ mental health experiences [42]. Universities should also strengthen partnerships with community-based organizations that specialize in support services for Asian communities. Expanding legal assistance for survivors with precarious immigration statuses and ensuring access to culturally specific healing practices—such as collective storytelling, intergenerational dialogue, and traditional wellness approaches—can significantly improve the campus climate for Asian female survivors [43]. 

Beyond service improvements, the findings call for a cultural shift in how universities address SVSH among Asian students. The model minority myth must be explicitly challenged in both research and campus policy, ensuring that the struggles of Asian survivors are neither ignored nor minimized. Universities should actively engage Asian student organizations and cultural groups to break the stigma around discussing sexual violence and mental health, fostering open and honest dialogue that empowers survivors to seek help without fear of judgment. The results also emphasized the need for faculty and staff training on the unique barriers that Asian female survivors face. Campus police, student affairs professionals, and Title IX coordinators should undergo culturally responsive training to better understand how issues like filial piety, family honor, and immigration fears impact survivors’ willingness to report and seek services. Without these considerations, many students will continue to be overlooked by institutional support systems, further reinforcing cycles of silence and trauma.

### Strengths and limitations

There were several limitations in the study worth discussion. Due to the use of convenience sampling, there was increased number of participants from three UC campuses: UC Los Angeles, Berkeley, and San Diego campuses. The available resources and campus culture may be different at relatively smaller institutions. Campus student clubs and organizations were contacted, primarily via social media, to spread awareness of the study. This may have limited how ethnic minority students were contacted and potentially led to selection bias. Furthermore, factors such as stigma and shame may have also prevented participation from Asian student-survivors that hold elevated stigma against SVSH, sex-related topics, and mental health. While members of the LGBTQAI + community were included in the study, further research is necessary to comparatively investigate the implications of the intersectionality of various sexual and gender identities on help-seeking behavior and access to resources.

A significant gap remains in understanding how the effects of SVSH vary across the diverse Asian diaspora and the implications for other minoritized groups. Enhancing campus prevention efforts and support resources requires further research into which approaches are most effective for students with intersecting identities. Future research can inform mandated education reforms that promote more equitable use of campus resources and improve student awareness and perceptions of available support. Overall, this study contributes to a growing understanding of how survivors’ sense of identity and belonging shapes their experiences of SVSH.

## Conclusion

The findings from this study highlight that addressing SVSH in Asian student populations requires a multidimensional approach that accounts for cultural, psychological, and structural barriers. By integrating culturally competent policies, expanding outreach to international students, and actively challenging racialized stereotypes, universities can create inclusive, survivor-centered environments where Asian female students feel empowered to seek support. Higher education institutions should proactively engage with these findings to ensure that campus policies reflect the realities of survivors’ experiences, fostering an academic climate that is safe, equitable, and responsive to the needs of all students.

## Supplementary Information

Supplementary Material 1.

## Data Availability

The datasets generated and analyzed during the current study are not publicly available due to confidentiality and ethical restrictions but are available from the corresponding author upon reasonable request.

## Footnotes

1 In this paper, the term “survivor” is used to describe individuals who have experienced sexual assault and/or harassment. Research has shown that this label rejects the narrative of oppression and victimhood and instead highlights strength and resistance.*.

2 In this paper, the term “survivor” is used to describe individuals who have experienced sexual assault and/or harassment. Research has shown that this label rejects the narrative of oppression and victimhood and instead highlights strength and resistance.***.
