# Supplementary material for: Navigating silence: cultural, familial, and immigration influences on the sexual violence experiences of Asian female college students in the university of California system
Source: BMC Public Health. 2025 Oct 1;25:3242. doi: 10.1186/s12889-025-24487-1 (PMC12486886; doi:10.1186/s12889-025-24487-1)
Supplement: Supplementary file 1 — Supplementary Material 1. [file 12889_2025_24487_MOESM1_ESM.docx]

**UNIVERSITY OF CALIFORNIA LOS ANGELES**

**Double Jeopardy Study Interview Guide**

**Welcome and Introductions:**

Hello (*participant’s name*). My name is ____________. I am (introduce yourself) and I am going to conduct this interview today. We can’t do this in person due to the pandemic, so thanks for joining me virtually today. I really appreciate your time. Let me tell you a little about the research project and what you can expect in this interview.

This interview is designed to gather information about your experiences as an ANHPI student Through the interview, I will be asking you about your observations and experiences with incidents of unwanted sexual contact and discriminations you may have experienced. The goal of the Double Jeopardy Study is to open the conversation about ANHPI students’ experiences of sexual violence, Xenophobia and discrimination before and during the pandemic, and use evidence-based practice to improve services to help students access supportive resources.

I hope you had read the information sheet we sent before. If not, we can review the Study Information Sheet now. [*Share your screen to review the information sheet.*]

The highlights of this interview are:

- We will meet for about 60 minutes.
- Participation in this interview is completely voluntary. You may decide to answer as many questions as you would like in as much or little detail as you are comfortable sharing. You may also decide not to answer questions. At any time, you are free to say you want to end the interview.
- I will be taking notes during the interview.
- I will be recording the interview even while I take notes because we don’t want to miss anything. You have an option to choose A) audio-only interview or B) video- and audio interview based on your preference. For transcription purposes, only voice will be recorded with your consent.
- All the information you provide will be kept confidential by the UCLA research team. Your name or other identifying information will not be shared with anyone, and will not be used in any reports that we write following this interview. If you would like to use a pseudonym during this interview, you may. If you want to use an alternative name, please introduce yourself to me with that name, or tell me what name you would like to use now.

You will receive a $50 gift card once we completed this interview.

Do you have any questions for me at this point? (answering questions if applicable)

Please say yes if you consent to participate in this study and to be recorded for the interview purposes.

[After consent] I will start recording on Zoom now, you will see a notification message showing on your screen.

**Semi-Structured Interview Questions**

**Section 1: Background**

Now, I would love to hear a bit about you and your background. *Prob: Can you describe your personal journey to this point (highlights- challenges, successes, milestones…)*

*Prob: What’s your family like?*

*Prob: How would you describe your life as a student?*

*(only for international students) Prob: How long have you been in the United States?*

*(only for international students) Prob: How’s your experience in the States so far?*

**Section 2: Discrimination & Sexual Violence Incidents**

Now I want to begin talking about your experience with potential **discrimination** as an Asian student.

1. What’s your perception of discrimination in the United States?
2. Can you give me an example of a time that you felt uncomfortable due to potential discrimination against your identify?
   1. How did that make you feel?
   2. What was your reaction?
3. How has it impacted your life?
   1. How has it impacted your academic life?
   2. How has it impacted your personal life?
   3. How has it impacted your mental or emotional health?

Now I’d like to talk about your experience with sexual violence. I just want to remind you that please let me know if you don’t feel comfortable sharing any of the details.

1. Can you give me an example of a time that you felt uncomfortable due to unwanted sexual contact?
   1. How often does this happen?
2. How does that make you feel?
3. How has it impacted your life?
   1. How has it impacted your academic life?
   2. How has it impacted your personal life?
   3. How has it impacted your mental or emotional health?

As you know, the Covid-19 pandemic has changed many things in this world…..

1. How has Covid-19 affected your life as an Asian student?
2. How do you think the pandemic has changed your experience of discrimination?
3. How do you think the pandemic has changed the experience of sexual violence?

**Section 3: Help-Seeking Behaviors and Coping Mechanisms**

I want to thank you for sharing your experience with us. Now I would like to understand more about how you coped with the incident(s).

1. How did you handle the incident?

2. What kind of support did you have?

3. What’s your understanding of on-campus services such as Title IX & CARE?

1. How would you think the involvement of these services may change the situation?
2. What kind of support did you wish you had back then?

**Section 4: Closing questions**

1. Now looking back to yourself as a survivor, can you describe something that you wish to change?
2. Is there something you may not have thought about before that occurred to you during our conversation?
3. Are there any more questions for me about this interview?
